# Lack of an Association between the Functional Polymorphism TREM-1 rs2234237 and the Clinical Course of Sepsis among Critically Ill Caucasian Patients—A Monocentric Prospective Genetic Association Study

**DOI:** 10.3390/jcm8030301

**Published:** 2019-03-03

**Authors:** Julius Runzheimer, Caspar Mewes, Benedikt Büttner, José Hinz, Aron-Frederik Popov, Michael Ghadimi, Katalin Kristof, Tim Beissbarth, Joel Schamroth, Mladen Tzvetkov, Bastian Schmack, Michael Quintel, Ingo Bergmann, Ashham Mansur

**Affiliations:** 1 Department of Anesthesiology, University Medical Center, Georg August University, D-37075 Goettingen, Germany; julius.runzheimer@med.uni-goettingen.de (J.R.); caspar.mewes@med.uni-goettingen.de (C.M.); benedikt.buettner@med.uni-goettingen.de (B.B.); katalin.kristof@med.uni-goettingen.de (K.K.); mquintel@med.uni-goettingen.de (M.Q.); ingo.bergmann@med.uni-goettingen.de (I.B.); 2 Department of Anesthesiology and Intensive Care Medicine, Klinikum Region Hannover, D-30459 Hannover, Germany; jose.hinz@krh.eu; 3 Department of Thoracic and Cardiovascular Surgery, University Medical Center, Eberhard Karls University, D-72076 Tuebingen, Germany; aronf.popov@gmail.com; 4 Department of General and Visceral Surgery, University Medical Center, Georg August University, D-37075 Goettingen, Germany; mghadim@uni-goettingen.de; 5 Institute of Medical Bioinformatics, University Medical Center, Georg August University, D-37077 Goettingen, Germany; tim.beissbarth@ams.med.uni-goettingen.de; 6 Department of Acute and General Medicine, Royal Free Hospital London, London NW3 2QG, UK; jlschamroth@gmail.com; 7 Department of Pharmacology, University Medical Center, Ernst-Moritz-Arndt-University, D-17487 Greifswald, Germany; mladen.tzvetkov@uni-greifswald.de; 8 Department of Cardiac Surgery, University Medical Center, Ruprecht-Karls-University, D-69117 Heidelberg, Germany; bastian.schmack@gmail.de

**Keywords:** 90-day mortality, sepsis, single nucleotide polymorphism, survival analysis, TREM-1

## Abstract

Sepsis is a life-threatening condition and a significant challenge for those working in intensive care, where it remains one of the leading causes of mortality. According to the sepsis-3 definition, sepsis is characterized by dysregulation of the host response to infection. The TREM-1 gene codes for the triggering receptor expressed on myeloid cells 1, which is part of the pro-inflammatory response of the immune system. This study aimed to determine whether the functional TREM-1 rs2234237 single nucleotide polymorphism was associated with mortality in a cohort of 649 Caucasian patients with sepsis. The 90-day mortality rate was the primary outcome, and disease severity and microbiological findings were analyzed as secondary endpoints. TREM-1 rs2234237 TT homozygous patients were compared to A-allele carriers for this purpose. Kaplan–Meier survival analysis revealed no association between the clinically relevant TREM-1 rs2234237 single nucleotide polymorphism and the 90-day or 28-day survival rate in this group of septic patients. In addition, the performed analyses of disease severity and the microbiological findings did not show significant differences between the TREM-1 rs2234237 genotypes. The TREM-1 rs2234237 genotype was not significantly associated with sepsis mortality and sepsis disease severity. Therefore, it was not a valuable prognostic marker for the survival of septic patients in the studied cohort.

## 1. Introduction

Sepsis is a life-threatening condition characterized by the severe dysregulation of the host response to infection [1]. It remains one of the leading causes of death among intensive care unit (ICU) patients [2]. There is no single standardized test or biomarker for diagnosing sepsis, which is particularly challenging when no causative infective organism is cultured [3]. The course of sepsis can be defined as a two-phase process: an infection leads to an overwhelming pro-inflammatory host response, which in turn leads to uncoordinated upregulation and downregulation of various inflammatory pathways (impacting on a wide range of cellular and intravascular processes) resulting in a dysregulated, weakened inflammatory response [4]. Patients often die in this secondary ‘anti-inflammatory’ phase [5]. This inadequate host response and the largely unexplored pathomechanisms provide potential targets for the future treatment of sepsis [6]. Studies suggest that the pro-inflammatory effect of the triggering receptor expressed on myeloid cells 1 (TREM-1) plays a key role in the host response to an infectious inflammation [7,8,9,10,11]. TREM-1 is expressed on neutrophils and macrophages associated with the signal transduction molecule DAP12 [7]. Due to the activation of the DAP12 signaling pathway, TREM-1 amplifies inflammatory response [12]. TREM-1 expression is enhanced by inflammatory stimuli such as lipopolysaccharides (LPS), a ligand for TLR4, or lipoteichoic acids [13,14]. It regulates the secretion of interleukin (IL)-8, tumor necrosis factor (TNF)-α and monocyte chemotactic protein-1 as well as granulocyte differentiation and degranulation [13,14]. Mice models suggest that blocking the TREM-1 signaling pathway positively affects the survival of mice with bacterial peritonitis and leads to lower levels of pro-inflammatory mediators [15]. Through the activation of blood neutrophils and monocytes, TREM-1 has an important effect on the acute host response and may therefore be a target for future novel therapies by regulating the neutrophil inflammatory responses [14,16].

As sepsis is a multifaceted syndrome complex of abnormal pathological, physiological and biochemical reactions induced by infection [1], the pathophysiology of the disease is diversely investigated, including analyses of a variety of polymorphisms, proteins and microRNA expression levels [17]. The genetic component of sepsis pathophysiology is polygenetic, as several single nucleotide polymorphisms (SNPs) have previously been associated with sepsis outcome and course of disease, which was also demonstrated in the prospectively enrolled population of this investigation [18,19,20,21].

The discovery of genetic variants that affect the expression and protein function of TREM-1 could be of diagnostic and therapeutic relevance. The present genetic association study examined the functional TREM-1 rs2234237 SNP that induces a change in the amino acid sequence and influences the biological function of TREM-1 [22]. The rs2234237 SNP has been shown to correlate with sepsis prognosis in Chinese Han septic patients [23] and to impact atherosclerosis and coronary artery disease risk as shown in a large Russian population [24]. Furthermore, a clinically relevant association of the rs2234237 T-allele with the development of pneumonia in mechanically ventilated burn patients was reported [25]. Thus, this polymorphism is supposed to have biological significance in terms of TREM-1 functionality and to impact on immunological pathways. Further to this, the present study aimed to assess whether the rs2234237 SNP is associated with sepsis prognosis and 90-day mortality in a large Caucasian cohort of patients with sepsis. Based on the reported findings of the TREM-1 rs2234237 T-allele as a risk factor for sepsis prognosis in a Chinese Han population and a higher susceptibility to pneumonia in mechanically ventilated burn patients, we hypothesized that carriage of the TT-genotype also predisposes for unfavorable sepsis mortality and disease severity in our prospectively enrolled cohort of Caucasian patients with sepsis.

## 2. Materials and Methods

### 2.1. Study Population

The prospectively enrolled patients in the present study were recruited from three surgical ICUs at the University Medical Center Goettingen. All patients were part of the GENOSEP database of the Department of Anesthesiology, comprising a large cohort of septic patients, their baseline characteristics (medical condition, premedication etc.) as well as relevant clinical parameters. The recruitment began in 2012, according to the valid sepsis definition at the time [1,26]. To ensure there was a homogeneous sample, only adult Caucasian patients were enrolled into the study. The following exclusion criteria were applied: pregnancy, immunosuppressing therapy or chemotherapy, history of heart attack within 6 weeks pre-enrollment, heart failure (New York Heart Association, NYHA IV), infection with human immunodeficiency virus (HIV), a pre-existing disease that would likely limit patient survival to less than 28 days, chronic vegetative state, patients already participating in an interventional study and patients who were study site employees or immediate family members of a study site employee [20]. No patients enrolled in this study were lost to follow-up.

This observational study was approved under the ethical project identification code 15/1/12 by the Institutional Ethics Committee of the University of Goettingen in Goettingen, Germany, and was performed in accordance with the provisions of the Declaration of Helsinki. The study was performed in accordance with relevant guidelines and regulations. The methods were performed in accordance with the approved guidelines. Written informed consent was obtained either from the patient or their legal representative.

### 2.2. Collection of Data

For patients that fulfilled the inclusion criteria, a blood sample was collected within 72 hours after sepsis onset. The baseline characteristics were collected, including pre-existing illnesses, surgical history, medications as well as the Acute Physiology and Chronic Health Evaluation (APACHE II) [27] and Sequential Organ Failure Assessment (SOFA) scores [28] of the day of sepsis onset. The analyzed clinical data were generated from the electronic patient record system (IntelliSpace Critical Care and Anesthesia (ICCA), Philips Healthcare, Andover, MA, USA). Daily SOFA scores were obtained for the length of stay (up to a maximum of 28 days after sepsis onset) to monitor organ failure. The need for organ support was recorded as a secondary outcome parameter. In order to gather the primary outcome parameters, a survival verification was obtained after 90 days [29].

All experimental protocols were performed in the laboratories of the Department of Clinical Pharmacology of the University Medical Center Goettingen. DNA extraction was performed in two different ways because a part of the blood samples was taken in ethylene diamine tetraacetic acid (EDTA) blood and another part was taken as peripheral blood mononuclear cells (PBMCs). For the EDTA blood extraction either a QIAamp^®^ DNA Blood Kit (Qiagen, Hilden, Germany) in a QIAcube^®^ or an EZ1^®^ DNA Blood Kit (Qiagen, Hilden, Germany) in a BioRobot EZ1^®^ was used. For the PBMC samples the extraction was performed with an AllPrep DNA Mini Kit (Qiagen, Hilden, Germany). Genotyping was performed via TaqMan polymerase chain reaction (PCR) with TaqMan^®^ SNP Genotyping Assay C__15948162_10 and a 7900HT Fast Real-Time PCR System (Life Technologies, Darmstadt, Germany). The quality and quantity of the DNA extraction was spectrophotometrically tested. The quality of the genotyping was verified by testing 15% of the samples in duplicate [30].

### 2.3. Data Analysis

The statistical analyses were performed with the STATISTICA 13 software (version 13.0, StatSoft, Tulsa, Oklahoma, USA). A *p*-value <0.05 was considered statistically significant. A log-rank-test was used for the Kaplan–Meier survival analysis. A multivariate Cox regression analysis was performed to exclude the effects of confounders on the survival. The Mann–Whitney-U-Test was performed for continuous variables while categorical variables were tested with a Pearson chi-square-test [30].

### 2.4. Availability of Data and Materials

The datasets used and analyzed during the current study are available from the corresponding author upon reasonable request.

## 3. Results

### 3.1. Baseline Characteristics at the Time of Enrolment

This study enrolled 649 septic patients from intensive care. The average age was 63 years with a standard deviation of 15 and ranged from 18 to 91. A total of 66% of the study population was male. The mean BMI was 28 with a standard deviation of 6. Furthermore, the sequential organ failure assessment (SOFA) and acute physiology and chronic health evaluation (APACHE II) scores were evaluated on day 1. The mean SOFA score was 9.4 ± 3.9 and the APACHE II score was 22 ± 7. The need for organ support at sepsis onset was 86% for mechanical ventilation, 67% for the use of a vasopressor and 9% for the need for dialysis. Mechanical ventilation during the observed period was needed by 93%, vasopressor use by 79% and dialysis by 21% of the patients. The mean length of stay was 20 ± 16 days.

The genotype distribution of TREM-1 rs2234237 was TT: *n* = 545, AT: *n* = 98 and AA: *n* = 6. This led to an observed minor allele frequency (MAF) of 0.096, almost equal to the expected MAF of 0.102 from the gnomAD—Exomes for a European population [31] reference population. The observed allele frequencies were consistent with the Hardy–Weinberg equilibrium (*p*-value: 0.4890).

We pooled the AT and AA genotypes in order to compare the TT homozygotes to the A-allele carries.

At baseline, the TT homozygous patients (*n* = 545) showed a significantly higher history of myocardial infarction (6% vs. 1%, *p* = 0.0290) compared to A-allele carriers (*n* = 104). Furthermore, at baseline, the TT homozygous patients’ recent surgical history (27% elective surgery, 54% emergency surgery, 19% no surgery) differed significantly from the A-allele carriers’ (40% elective surgery, 49% emergency surgery, 11% no surgery, *p* = 0.0103). All baseline characteristics data can be obtained from Table 1.

As a measure of organ dysfunction, the necessity of organ support (mechanical ventilation, use of vasopressors and renal replacement therapy) on sepsis onset and during observation period was studied, but did not reveal significant differences.

### 3.2. Mortality Analysis

**Figure 1:**
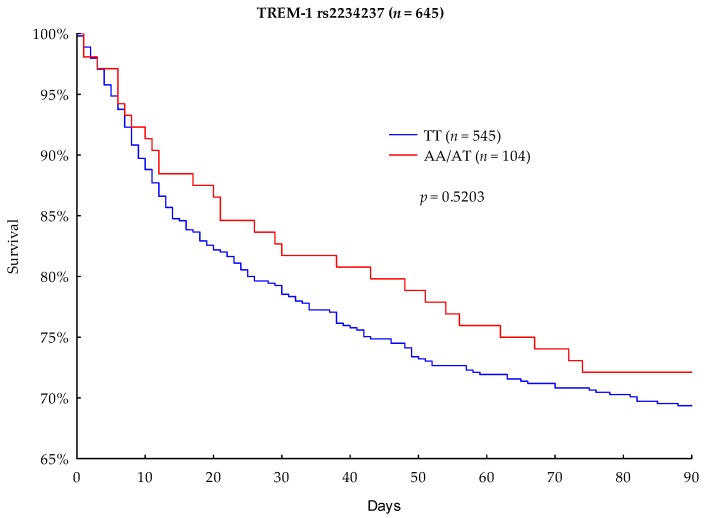
Kaplan–Meier 90-day survival analysis with regard to TREM-1 rs2234237 genotypes.

**Figure 2:**
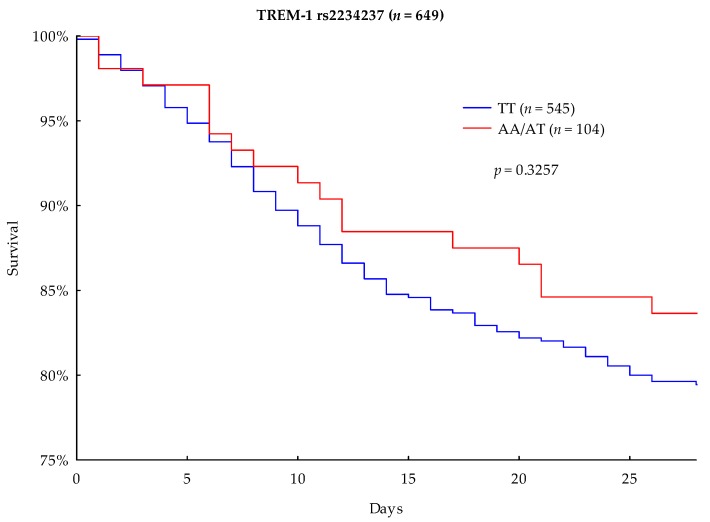
Kaplan–Meier 28-day survival analysis with regard to TREM-1 rs2234237 genotypes.

The primary outcome parameter was 90-day mortality. In the performed Kaplan–Meier survival analysis, A-allele carriers showed a lower mortality rate than the TT homozygous carriers (27.88% vs. 30.64%, respectively); however, the result was not significant (*p* = 0.5203) (Figure 1). The 28-day survival analysis showed that A-allele carriage was associated with a lower mortality rate than TT homozygotes (16.35% vs. 20.55%), but this difference did not achieve statistical significance (*p* = 0.3257) (Figure 2).

### 3.3. Multivariate Analysis

In order to recognize the effects of the potential confounders and variables of the baseline characteristics on the 90-day mortality, we performed a multivariate Cox regression analysis. Age, body mass index (BMI), SOFA score at day 1, APACHE II score at day 1 and male gender were included as baseline variables. The presence of myocardial infarction as pre-existing medical condition was included as a potential confounder due to the results of the patient baseline characteristics. Our results show that age, SOFA score day 1 and APACHE II score day 1 had a significant impact on survival. In contrast, the TREM-1 rs2234237 TT-genotype did not significantly impact upon survival (hazard ratio: 1.11, 95% CI: 0.74–1.65, *p*-value = 0. 6098 (Table 2)).

### 3.4. Disease Severity

In order to evaluate the association between the TREM-1 rs2234237 genotypes and disease severity, the authors examined the SOFA scores, organ-specific SOFA sub-scores, organ support and various hematological and biochemical parameters. Organ support was divided in three subgroups: ventilation-free days, vasopressor-free days and dialysis-free days. The analyzed blood tests were leucocytes, C-reactive protein (CRP), and procalcitonin, as well as parameters for liver function, kidney function and coagulation. The observation period was 28 days after sepsis onset, unless the patient was discharged prior to this or deceased.

We found no significant differences in disease severity between TREM-1 rs2234237 genotypes (Table 3).

Furthermore, the observed microbiological findings were compared, but also revealed no significant differences.

## 4. Discussion

The TREM-1 receptor on neutrophils and macrophages has been shown to have a pro-inflammatory effect on the host response to infection. Previous studies have demonstrated that the blocking of TREM-1 signaling is associated with a reduction of inflammatory mediators such as IL-1, TNF-α, Monocyte chemoattractant protein 1 (MCP-1) and interferon (IFN)-γ and prolonged survival in mice with bacterial infection [15]. Considering the need for novel diagnostic approaches in the treatment of sepsis [4,32], other studies discussed the role of soluble TREM-1 as a potential biomarker in sepsis [33]. The aim of our study was to investigate the impact of a genetic variant of the TREM-1 gene, which is located on chromosome 6, on the survival of patients with sepsis. We examined the functional TREM-1 rs2234237 SNP, a variation in exon 2 of the TREM-1 gene.

An accordance with the a priori hypothesis, this study found that TREM-1 rs2234237 TT-genotype carriers had an unfavorable 90- and 28-day survival rate compared to A-allele carriers (30.64% vs. 27.88%). However these results were not statistically significant (*p* = 0.5203). Furthermore, the performed multivariate Cox regression analysis did not significantly show that the TREM-1 rs2234237 TT-genotype is a valuable prognostic marker for the survival of septic patients. According to the Cox regression analysis, the effect of the TREM-1 rs2234237 TT genotype on 90-day survival could be in the range of −25.5% to +65.2% (hazard ratio 95% CI: 0.7448–1.6522), which was however not significantly detected in our cohort (*p* = 0.6098). Our findings are consistent with previous studies in Chinese Han populations, which also did not reveal a significant impact of the TREM-1 rs2234237 genotype on the 28-day outcome of patients in septic shock [22] or the development of severe sepsis [34]. However, different population allele frequencies and a divergent study design needed to be considered in these studies.

As secondary endpoints, the patient baseline characteristic and disease severity were examined with regard to the TREM-1 rs2234237 genotypes. These analyses showed that A-allele carries had a significantly lower history of myocardial infarction compared to TT homozygous patients. Moreover, A-allele carriers differed in their recent surgical history, and had significantly more elective surgeries and less emergency surgeries as well as less conservative treatments as the reason for recent consultations. These findings suggest that TREM-1 may play a role in a range of disease processes, and this is certainly an area that requires further exploration and investigation. We supposed that the functional TREM-1 rs2234237 SNP that induces a change in the amino acid sequence and influences the biological function of TREM-1 [22] might affect sepsis mortality and disease severity in a relatively large cohort of prospectively enrolled septic Caucasian patients. However, our a priori hypothesis could not be verified by the performed investigations and thus needs to be rejected or possibly studied in other cohorts or with different study designs. The results do not allow us to draw conclusions regarding the contribution of the TREM-1 molecule to the pathophysiology of the disease. Possible alterations of TREM-1 functionality may not be relevant to the etiology and pathophysiology of the disease in the studied cohort. To further investigate alterations in functionality, TREM-1 protein expressions (i.e., soluble TREM-1), RNA expressions or TREM-1 protein function should be studied as direct measures of pathophysiology in sepsis.

As this study is a genetic association study, multiplicity, population stratification and inadequate sample size need to be acknowledged as potential limitations. While a well-defined prospectively enrolled homogeneous cohort of patients is certainly a strength of our investigation, the monocentric design and focus on Caucasian ethnicity need to be considered as limitations. The study was conducted in a single center and findings therefore need to be further validated in independent cohorts from other centers to assess their generalizability, also including different ethnicities. Moreover, the additive effects of other SNPs in the TREM-1 gene—such as rs2234246, rs7768162 or rs9471535—on sepsis outcome have not been investigated in this study, but need to be considered. Furthermore, a power calculation to determine a sample size with adequate power at the beginning of the investigation could not be conducted, because the effect of the TREM-1 rs2234237 polymorphism on 90-day mortality in patients with sepsis was unknown. Though, we assumed that the relatively rare genetic variant rs2234237 (MAF of 0.102; according to gnomAD—Exomes for a European population) needs to show an effect of at least 15% mortality reduction in our cohort of 649 septic Caucasians in order to act as a prognostic marker for survival in patients with sepsis. Such an effect of 15% mortality reduction would have shown a power of 0.911. Accordingly, the observed slight effect of 2.76% mortality reduction (27.88% vs. 30.64% for A-allele carriers and TT homozygous carriers, respectively) in a representative cohort of 649 septic Caucasians means that the polymorphism rs2234237 cannot act as a useful predictor of survival in routine clinical practice. In addition, our analysis of disease severity was performed in a univariate model and not adjusted in a multivariate analysis.

## 5. Conclusions

To the best of the authors’ knowledge, this is the first study investigating the effects of the TREM-1 rs2234237 genotype on the mortality and disease severity of Caucasian patients with sepsis. In conclusion, the TREM-1 rs2234237 genotype is not significantly associated with sepsis mortality or disease severity.

## Figures and Tables

**Table 1:** Patient baseline characteristics with regard to TREM-1 rs2234237 genotypes.

	All (*n* = 649)	AA/AT (*n* = 104)	TT (*n* = 545)	*p*-Value
General				
Age in years	63 ± 15	64 ± 14	63 ± 15	0.5392
Sex: Male (%)	66	66	66	0.9254
BMI	28 ± 6	27 ± 6	28 ± 6	0.9725
Severity				
SOFA Day 1	9.4 ± 3.9	9.0 ± 4.1	9.4 ± 3.8	0.1421
APACHE II Day 1	22 ± 7	21 ± 7	22 ± 7	0.3042
Septic shock (%)	51	57	50	0.1800
Days in septic shock	2 ± 3	2 ± 3	2 ± 3	0.1453
Procalcitonin ng/dl Day 1	10 ± 29	10 ± 26	10 ± 30	0.8157
Comorbidities (%)				
Hypertension	54	50	55	0.3804
History of myocardial infarction	5	1	6	0.0290
Chronic obstructive pulmonary disease	15	14	15	0.8703
Bronchial asthma	3	2	3	0.6275
Renal dysfunction	10	11	10	0.9262
Noninsulin-dependent diabetes mellitus	9	11	8	0.4805
Insulin-dependent diabetes mellitus	10	11	10	0.9712
Chronic liver disease	6	5	7	0.4898
History of cancer	15	20	15	0.2387
History of stroke	6	4	6	0.3410
Dementia	3	4	3	0.7800
Rheumatoid arthritis	1	0	1	0.2824
Recent surgical history (%)				0.0103
Elective surgery	29	40	27	
Emergency surgery	53	49	54	
No surgery	18	11	19	
Organ support during observation period (%)				
Mechanical ventilation	93	92	94	0.6332
Use of vasopressor	79	78	79	0.7186
Renal replacement therapy	21	18	21	0.5133
Organ support on sepsis onset (%)				
Mechanical ventilation	86	87	86	0.8200
Use of vasopressor	67	64	67	0.5878
Renal replacement therapy	9	8	9	0.6681
Length of stay:				
LOS ICU	20 ± 16	21 ± 17	20 ± 16	0.7486

Footnote: SOFA, sequential organ failure assessment; APACHE, acute physiology and chronic health evaluation; LOS, Length of stay; ICU, Intensive care unit; BMI, body mass index.

**Table 2:** Cox regression analysis (90 days) with regard to TREM-1 rs2234237 genotypes.

Variable	Hazard Ratio	95% CI	*p*-Value
Age	1.0270	1.0150–1.0391	0.0000
Body mass index	0.9795	0.9544–1.0053	0.1181
SOFA score	1.0704	1.0228–1.1203	0.0034
APACHE II score	1.0335	1.0046–1.0633	0.0226
Male gender	1.0481	0.7771–1.4137	0.7582
Myocardial infarction	1.2061	0.6956–2.0913	0.5046
TREM-1 rs2234237 TT genotype	1.1093	0.7448–1.6522	0.6098

Footnote: SOFA, sequential organ failure assessment; APACHE, acute physiology and chronic health evaluation.

**Table 3:** Disease severity with regard to TREM-1 rs2234237 genotypes.

	All (*n* = 649)	AA/AT (*n* = 104)	TT (*n* = 545)	*p*-Value
General				
SOFA	7 ± 4	7 ± 4	7 ± 3	0.4717
SOFA respiratory score	2 ± 1	2 ± 1	2 ± 1	0.8800
SOFA cardiovascular score	2 ± 1	2 ± 1	2 ± 1	0.7582
SOFA central nervous system score	2 ± 1	2 ± 1	2 ± 1	0.1310
SOFA renal score	1 ± 1	1 ± 1	1 ± 1	0.8692
SOFA coagulation score	0 ± 1	0 ± 1	0 ± 1	0.2439
SOFA hepatic score	0 ± 1	0 ± 1	0 ± 1	0.8126
Organ support free days				
Ventilation free days	5 ± 5	4 ± 5	5 ± 5	0.8157
Vasopressor free days	10 ± 7	10 ± 7	10 ± 7	0.5074
Dialysis free days	14 ± 8	14 ± 8	14 ± 8	0.7018
Ventilation free days/observation days (%)	66 ± 32	65 ± 34	67 ± 31	0.9150
Vasopressor free days/observation days (%)	34 ± 30	34 ± 30	33 ± 30	0.7081
Dialysis free days/observation days (%)	9 ± 22	9 ± 22	10 ± 23	0.6751
Inflammatory values:				
Leucocytes (1000/µL)	13 ± 5	13 ± 5	13 ± 5	0.4353
CRP (mg/L)	151 ± 87	164 ± 89	148 ± 86	0.1946
Procalcitonin (ng/dL)	4 ± 9	5 ± 13	4 ± 8	0.9562
Kidney values				
Urine output (ml/day)	2980 ± 1333	2864 ± 1280	3002 ± 1343	0.2934
Urine output (ml/kg/h)	2 ± 1	2 ± 1	2 ± 1	0.7470
Creatinine (mg/dL)	1.2 ± 1	1.2 ± 0.8	1.2 ± 1	0.7346
Liver values				
AST (GOT) (IU/L)	168 ± 595	114 ± 229	177 ± 642	0.3071
ALT (GPT) (IU/L)	93 ± 188	74 ± 142	97 ± 195	0.0630
Bilirubin (mg/dL)	1.2 ± 2	1.1 ± 1.7	1.2 ±2	0.9725
Central nervous system				
GCS	10 ± 3	10 ± 3	10 ± 3	0.1553
Coagulation:				
Thrombocytes (1000/µL)	296 ± 152	274 ± 131	300 ± 155	0.1810

Footnote: SOFA, sequential organ failure assessment; CRP, C-reactive protein; GCS, Glasgow Coma Scale; AST, aspartate amino transferase; GOT, glutamic oxalo acetic transaminase; ALT, alanine aminotransferase; GPT, glutamic-pyruvic transaminase.
